# Genetic improvement of *n*-butanol tolerance in *Escherichia coli* by heterologous overexpression of *groESL* operon from *Clostridium acetobutylicum*

**DOI:** 10.1007/s13205-014-0235-8

**Published:** 2014-07-17

**Authors:** Ali S. Abdelaal, Amr M. Ageez, Abd El-Hadi A. Abd El-Hadi, Naglaa A. Abdallah

**Affiliations:** 1 Agricultural Genetic Engineering Research Institute (AGERI), Agricultural Research Center (ARC), Giza, Egypt; 2 Genetics Department, Faculty of Agriculture, Cairo University, Giza, Egypt

**Keywords:** Heat shock protein, *groESL*, *n*-Butanol, Solvent tolerance, Biofuels

## Abstract

Strain tolerance to toxic metabolites remains an important issue in the production of biofuels. Here we examined the impact of overexpressing the heterologous *groESL* chaperone from *Clostridium acetobutylicum* to enhance the tolerance of *Escherichia coli* against several stressors. Strain tolerance was identified using strain maximum specific growth rate (*μ*) and strain growth after a period of solvent exposure. In comparison with control strain, the *groESL* overexpressing strain yielded a 27 % increase in growth under 0.8 % (v/v) butanol, a 9 % increase under 1 % (v/v) butanol, and a 64 % increase under 1.75 (g/l) acetate. Moreover, after 10 h, *groESL* overexpression resulted in increase in relative tolerance of 58 % compared with control strain under 0.8 % (v/v) butanol, 56 % increase under 1 % (v/v) butanol, 42 % increase under 1 % (v/v) isobutanol, 36 % increase under 4 % (v/v) ethanol, 58 % increase under 1.75 (g/l) acetate. These data demonstrate that overexpression of the *groESL* from *C. acetobutylicum* in *E. coli* increased tolerance to several stressors. Solvent tolerant strain of *E. coli* was developed to be used as a basic strain for biofuel production.

## Introduction

Concerns about the global energy crisis, coupled with increased awareness of global warming, have spurred an interest in developing alternatives to fossil fuels. Due to their renewable features, biofuels are potential candidates for partially, or completely, replacing crude oil. Presently, ethanol fermented from starch or sugar is the most widely used biofuel due to the ease of manufacturing it from agricultural feedstock. Meanwhile, there is increasing interest in butanol as an advanced alternative biofuel with several distinctive advantages over ethanol based on a number of attractive attributes, including its higher energy density, miscibility with gasoline, higher octane rating, lower volatility, lower vapor pressure, less corrosive and less water solubility (Connor and Liao 2009). Typically, biobutanol can be produced by acetone–butanol–ethanol (ABE) fermentation using anaerobic bacteria, i.e. Clostridia.

The toxic nature of solvents on bacteria is a major limiting factor in the production of chemicals by fermentation (Isken and de Bont 1998). Accumulation of organic solvents has been shown to permeabilize the cell membrane, resulting in a passive flux of ATP, protons, ions, and macromolecules such as RNA and proteins (Sikkema et al. 1995). Solvents may also disrupt the function of embedded membrane proteins and drastically alter membrane fluidity (Bowles and Ellefson 1985; Sikkema et al. 1994). Growth has been shown to be the most sensitive cellular activity to the effects of solvents (Ingram 1990).

Butanol toxicity/inhibition to the fermenting microorganisms is one of the major barriers currently facing the production of biobutanol. Even the native producer, *Clostridium acetobutylicum*, only tolerates up to 1–2 % (v/v) of this organic solvent (Winkler et al. 2010), resulting in a low butanol titer in the fermentation broth. The toxicity of butanol in *C. acetobutylicum* is quite severe, and this has been attributed to its chaotropic effect on the cell membrane (Bowles and Ellefson 1985; Vollherbst-Schneck et al. 1984). High concentrations of butanol have inhibition effects on nutrient transport, membrane-bound ATPase activity and glucose uptake (Bowles and Ellefson 1985). *C. acetobutylicum* fermentations rarely produce butanol higher than 13 g/L, a level that is inhibitory for the growth of *C. acetobutylicum* and is generally considered as the toxic limit (Jones and Woods 1986). Butanol is the most toxic produced solvent to *C. acetobutylicum* as it reduces cell growth by 50 % at a concentration of 7–13 g/L (Tomas et al. 2003; Lee et al. 2008). Economic analysis of butanol fermentation indicates that even a slight increase of the *n*-butanol concentration in the fermentation broth would reduce separation costs and leads to an economically viable process (Papoutsakis 2008), dictating the scientific community to engineer microbes for increased butanol tolerance.

Although *C. acetobutylicum* has been used as a natural butanol producer for decades, it has several drawbacks, such as a slow growth rate, complex regulatory pathways, and difficulties in genetic manipulation (Jeong and Han 2010). In response to this, *Escherichia coli* has been metabolically engineered as an alternative host for butanol production by introducing a butanol production pathway (Atsumi et al. 2008a; Nielsen et al. 2009), due to its well-characterized genetic background and well-developed genetic tools, allowing for a flexible and economical process design for large-scale production. In order for this microorganism to produce biobutanol viably, it must be able to survive under certain concentration of this biofuel. Unfortunately, *E. coli* growth is severely inhibited by butanol, being almost completely stopped by 1 % (v/v) butanol (Atsumi et al. 2008b). This lack of butanol tolerance of *E. coli* has spurred research on the development of *E. coli* strains with improved butanol tolerance.

Most organisms with demonstrated ability to tolerate otherwise toxic solvent levels have cellular adaptations which effectively suppress solvent effects on the membrane through changes in membrane composition (Isken and de Bont 1998). Another class of solvent-tolerant bacteria includes those with an efflux system, which actively decreases the concentration of toxic solvents within the cell (Ramos et al. 2002). A third mechanism, similar to that of antibiotic resistance, is degradation of the toxic substance to a less toxic product (Ferrante et al. 1995). Finally, toxic solvents have been shown to induce known stress (heat shock) proteins (HSPs). The ubiquitous heat shock proteins, also called molecular chaperones, the primary members of the general stress response system, play an essential role in the folding and transport of proteins, as well as remediation of damaged or misfolded proteins (Zingaro and Papoutsakis 2012a). Solventogenic phase and butanol-stressed clostridia express stress genes, including all major chaperones (Alsaker and Papoutsakis 2005).

The first aim of this study was to characterize the physiological response of *E. coli* to exogenous *n*-butanol, isobutanol, ethanol, and acetate stressors. Based upon the previous work in *C. acetobutylicum*, whereby *groESL* overexpression provided tolerance to butanol stress, the second aim was to evaluate the potential influence of heterologous overexpression in *E. coli* with heat shock protein (*groESL*) from *C. acetobutylicum* to exogenous *n*-butanol and other stressors. The last aim was to develop butanol tolerant strain of *E. coli* to render it more suitable, and can be used as a basic strain for butanol production.

## Materials and methods

### Bacterial strains and plasmids

The *C. acetobutylicum* ATCC 824 and the *E. coli* DH10β and BL21 strains were used in this study. pGEM^®^-T Easy and pF1A T7 Flexi vectors (Promega, Madison, WI, USA) were used for cloning and overexpression studies.

### Growth conditions and maintenance

*C. acetobutylicum* strain was grown in an anaerobic chamber (Forma Scientific, Marietta, OH, USA) at 37 °C in clostridia growth medium (CGM). Single colony, at least 5 days old, was obtained from agar-solidified medium (Lab M, UK) and used to inoculate liquid culture for growth at 37 °C. *E. coli* strains were grown aerobically in liquid Luria–Bertani (LB) medium at 10 RCF (New Brunswick Scientific, NJ, USA) and 37 °C, and on agar-solidified LB at 37 °C. When required, the medium was supplemented with the antibiotic: ampicillin at 100 mg/ml. Frozen stocks were prepared from overnight cultures and stored in LB plus 17.5 % glycerol at −80 °C. Cells from a single colony were used to inoculate liquid cultures. Growth curves were carried out in M9 minimal media supplemented with 5 g/L of glucose, supplemented with ampicillin.

### Sequence adjustment

Sequences of bacterial co-chaperonin *groES* and chaperonin *groEL* genes from *C. acetobutylicum* ATCC 824 and *E. coli* were obtained from the NCBI non-redundant and dbEST data sets using BLASTP (ver. 2.2.28+) (Altschul et al. 1997). The full amino acid sequences of the proteins were compositionally adjusted using compositional score matrix adjustment.

### DNA isolation and transformation

Isolation of genomic DNA from *C. acetobutylicum* ATCC 824 strain was performed using the Wizard^®^ Genomic DNA Purification Kit (Madison, USA, USA). Transformations were carried out with DH10β chemically competent cells for cloning construct and electroporation was used in expression constructs in BL21 electro-competent cells.

### Gene cloning and sequence analysis

Oligonucleotide primers “Cac-groESL-F and Cac-groESL-R” with the sequences of 5′-GCCAAAATTAAGTTTATACTAAAAG-3′ and 5′-AATGCACTCTTATTACATTAATC-3′ respectively (Tomas et al. 2003), were used to amplify *groESL* operon. The *groESL* operon was PCR amplified using the primers Cac-groESL-F and Cac-groESL-R with the *C. acetobutylicum* chromosomal DNA as a template. The product was then cloned into a linearized pGEMTeasy vector and chemically transformed into DH10β competent cells. Isolation of plasmid DNA from *E. coli* was performed using the Zyppy™ Plasmid Miniprep Kit (Zymo, USA). The recombinant clone was sequenced using a Big Dye Terminator Cycle Sequencing FS Ready Reaction Kit (Applied Biosystems, Foster City, CA, USA). A homology search was performed using BLASTN against the NCBI nucleotide database (http://www.ncbi.nlm.nih.gov).

### Plasmid construction

**Fig. 1:**
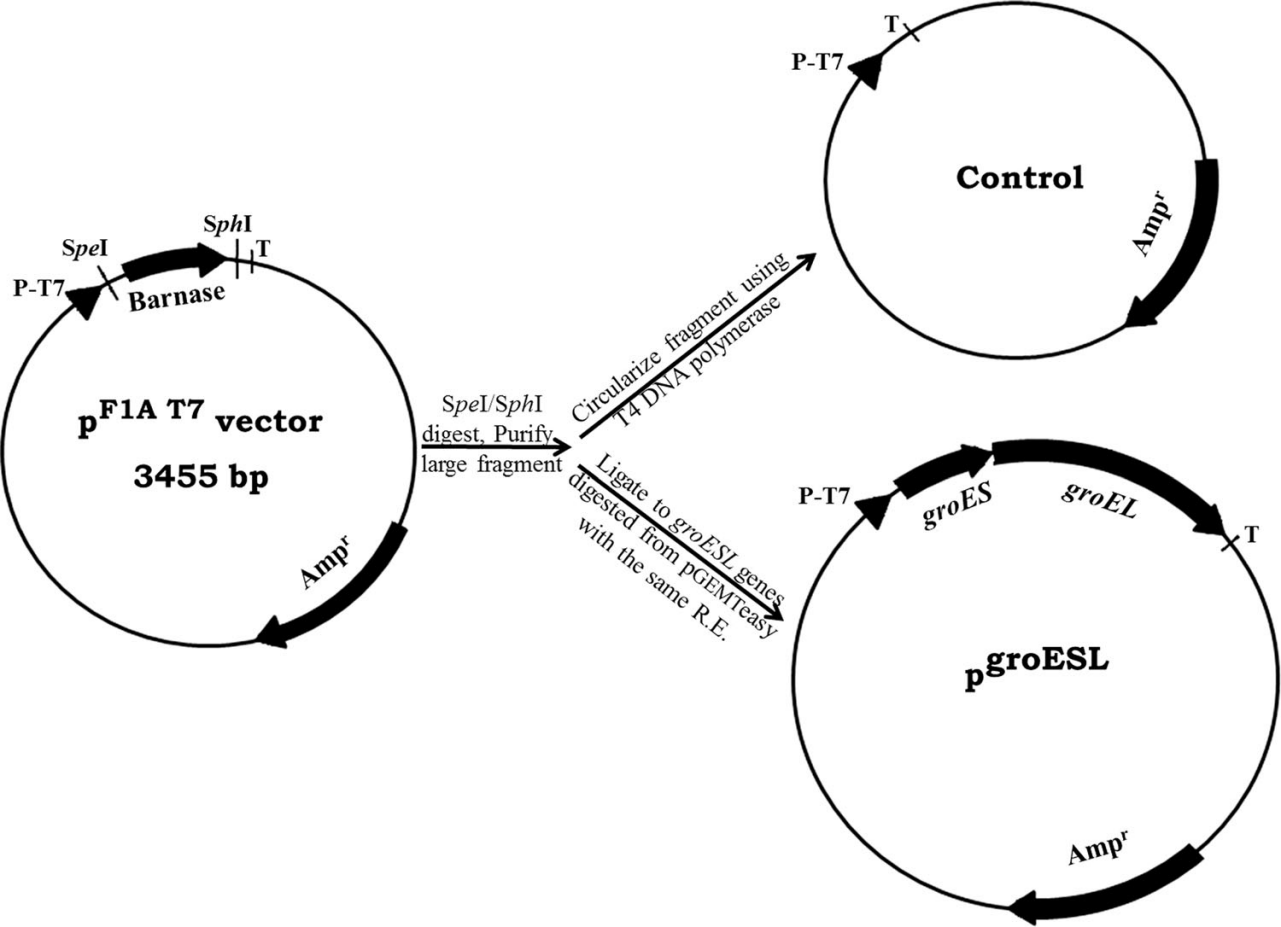
Construction of pF1A T7 Flexi (−) and pgroESL. The location and direction of relevant genes are indicated with *arrows*. Relevant restriction sites are shown

The plasmid pCac-groESL was designed to overexpress the *C. acetobutylicum* *groES* and *groEL* genes forming *groESL* operon under T7 regulatory elements (promoter). According to the direction of the *groESL* operon in pGEMTeasy vector and the restriction sites in pGEMTeasy and pF1A T7 Flexi vectors, the *groESL* was double digested from pGEMTeasy vector using *Spe*I and *Sph*I and ligated into pF1A T7 Flexi vector digested with the same restriction enzymes as shown in Fig. 1. This plasmid was then transformed into BL21 electro-competent cell for gene expression (Transformed strain). Oligonucleotide primers “Flexi-F and Flexi-R” with the sequences of 5′-AGGGGAATTGTGAGCGGATAA-3′ and 5′-CTCAGCTTCCTTTCGGGCTT-3′, respectively, were designed using Primer3 and BLAST. The recombinant clone was sequenced using Flexi primers to confirm the direction of the *groESL* operon. For control strain, the pF1A T7 Flexi vector was double digested using *Spe*I and *Sph*I, converted to blunt-ends DNA using T4 DNA polymerase and self-ligated to eliminate Barnase lethal gene and forming pF1A T7 Flexi (−) as shown in Fig. 1, and then transformed into BL21 electro-competent cell.

### Butanol challenge experiment

groESL transformed strain was cultured in M9 minimal media (5 g/L glucose) and incubated overnight at 37 °C to be used as inoculum. On the next day, a 5 % (v/v) inoculum was used to seed a 30 mL culture in 250 mL closed-cap flasks for growth kinetic analysis in the absence and presence of 0.8 and 1 % (v/v) *n*-butanol. Three biological replicas were obtained per sample. Bacterial growth was monitored using spectrophotometry (optical density at 600 nm [OD600]) until stationary phase was reached. The growth kinetic parameter “*s*” described below was calculated. Statistical significance was assessed using a Student’s *t* test analysis using a *p* value cut-off of 0.05. Standard deviation was used to measure the amount of variation from the average.

### Calculation of growth kinetic parameters

The growth kinetics parameters: “percentage of inhibition”, “relative fitness coefficient (s)” and “relative increase in fitness (RIF)” were calculated using Eqs. (1), (2) and (3), respectively (Reyes et al. 2013). These parameters were calculated using the measured maximum specific growth rate (*µ*_i_) of each strain (strain *i*).

1 $${\text{Inhibition}} \, (\% ) = \left[ {1 - \left( {\frac{{\mu_{{{\text{clone}}\, \,@\,\, {\text{stressful condition}}}} }}{{\mu_{{{\text{clone in absence of}}\;{\text{stressor}}}} }}} \right)} \right] \times 100$$

2 $$s \, (\% ) = \left[ {\left( {\frac{{\mu_{\text{clone @ stressful condition}} }}{{\mu_{\text{reference strain @ stressful condition}} }}} \right) - 1} \right] \times 100$$

3 $${\text{RIF}} \, (\% ) = \left[ {1 - \left( {\frac{{{\text{Inhibition}}_{\text{clone @ stressful condition}} }}{{{\text{Inhibition}}_{\text{reference strain @ stressful condition}} }}} \right)} \right] \times 100$$

Other growth kinetics parameters, “percentage of tolerance” and “relative tolerance (RT)”, were calculated using Eqs. (4) and (5), respectively (Borden and Papoutsakis 2007). These parameters were calculated using the measured growth after a period of time.

4 $${\text{Tolerance}} \,(\% ) = \frac{{A_{{ 6 0 0 {\text{\% challenge, }}\,t }} - A_{{ 6 0 0 {\text{\%}}\;{\,\text{challenge, }}\,t_{ 0} }} }}{{A_{{600 {\text{ no challenge}},\, t }} - A_{{600 {\text{ no challenge}},\, t_{0} }} }} \times 100$$

5 $${\text{RT}} \,(\% ) = \left[ {1 - \left( {\frac{{{\text{Tolerance}}_{\text{clone @ stressful condition}} }}{{{\text{Tolerance}}_{\text{reference strain @ stressful condition}} }}} \right)} \right] \times 100$$

### Phenotypic analysis of *n*-butanol-tolerance conferring *groESL* gene

Transformed strain that showed a statistically significant increase in relative fitness in the presence of *n*-butanol was validated in batch cultures under other stressors. The stressors analyzed in this study were 0.8 % (v/v) *n*-butanol, 1 % (v/v) *n*-butanol, 1 % (v/v) isobutanol, 4 % (v/v) ethanol, 1.75 g/L of acetate. Cultures were incubated at 37 °C with constant shaking at 10 RCF.

## Results and discussion

The aim of this study was to develop butanol tolerant strain of *E. coli* that can be used as a basic strain for butanol production by means of overexpression of heat shock protein, *groESL* isolated from *C. acetobutylicum.* Previous studies used *groESL* isolated from *E. coli* for autologous overexpression and showed increasing in butanol tolerance.

### Amino acids sequences producing significant alignments

**Fig. 2:**
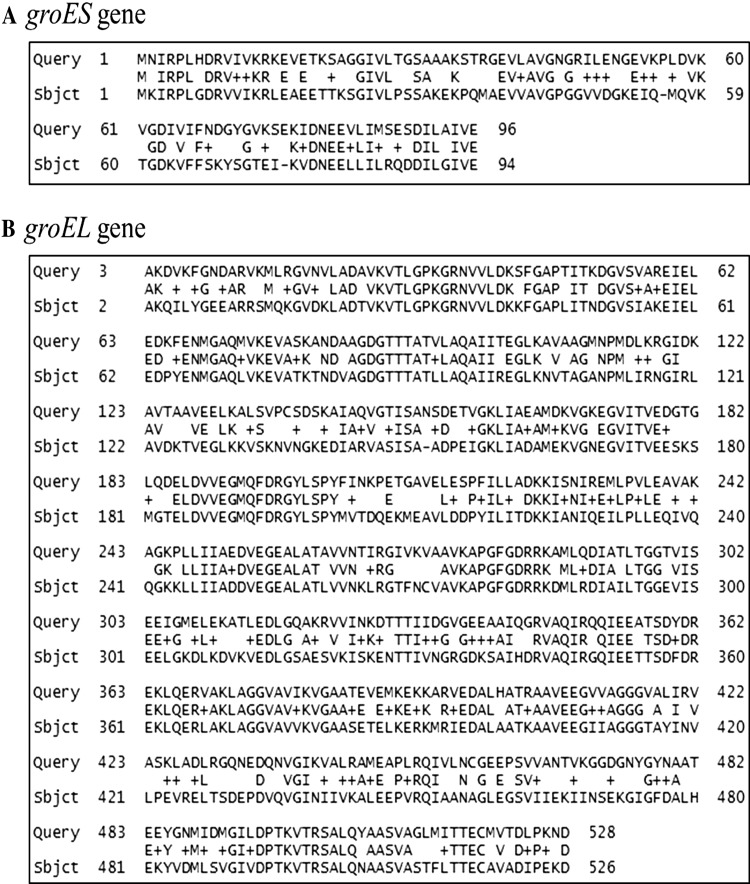
Amino acid sequences alignment of *groES* and *groEL* genes

Autologous overexpression of *groESL* in *C. acetobutylicum* and *E. coli* were performed and increased solvent tolerance (Tomas et al. 2003; Zingaro and Papoutsakis 2012b). To determine the identities of co-chaperonin *groES* and chaperonin *groEL* from *C. acetobutylicum* ATCC 824 to co-chaperonin *groES* and chaperonin *groEL* from *E. coli*, the compositional score matrix adjustment was used to align amino acid sequence homology. The alignments showed that the identity of *groES* protein from *C. acetobutylicum* ATCC 824 and *E. coli* was 48 % and the identity of *groEL* protein from *C. acetobutylicum* ATCC 824 and *E. coli* was 61 % as shown in Fig. 2. The low identities in amino acid sequences increased the possibility of a significant effect of heterologous overexpression of *groESL* from *C. acetobutylicum* to *E. coli.*

### Isolation of *groESL* from *C. acetobutylicum*

**Fig. 3:**
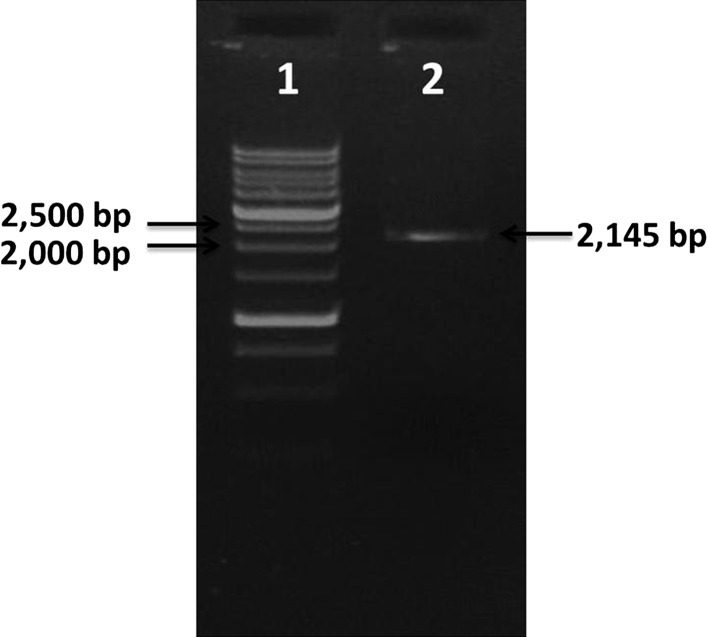
Amplification of *groESL* operon. *Lane 1*. 1 kb ladder, *Lane 2*. *groESL* operon (2145 bp)

Total DNA isolated from *C. acetobutylicum* was used to amplify *groESL* operon using specific primers. *groESL* specific primers were used to amplify an operon of 2,145 bp (Fig. 3). The amplified *groESL* was purified, cloned into the pGEM^®^-T Easy vector, transformed into DH10β strain and sequenced. The sequence was confirmed by BLASTN.

### Cloning of *groESL* operon in pF1AT7 Flexi vector

Both *groESL* operon into the pGEMT-easy vector and pF1AT7 Flexi vector were double digested with *Spe*I and *Sph*I restriction enzymes. The restriction enzymes were selected using NEBcutter, so they do not cut into the operon and to ensure the right orientation of the *groESL* operon in pF1A T7 Flexi vector. The digested *groESL* operon was ligated into digested pF1AT7 Flexi vector. The *groESL* and control clones were separately transformed into BL21 strain and confirmed using PCR test. The *groESL* operon was sequenced using Flexi-F and Flexi-R oligonucleotide primers.

### *groESL* heterologous overexpression under T7 promoter imparts higher butanol tolerance to *E. coli* and other stressors

**Fig. 4:**
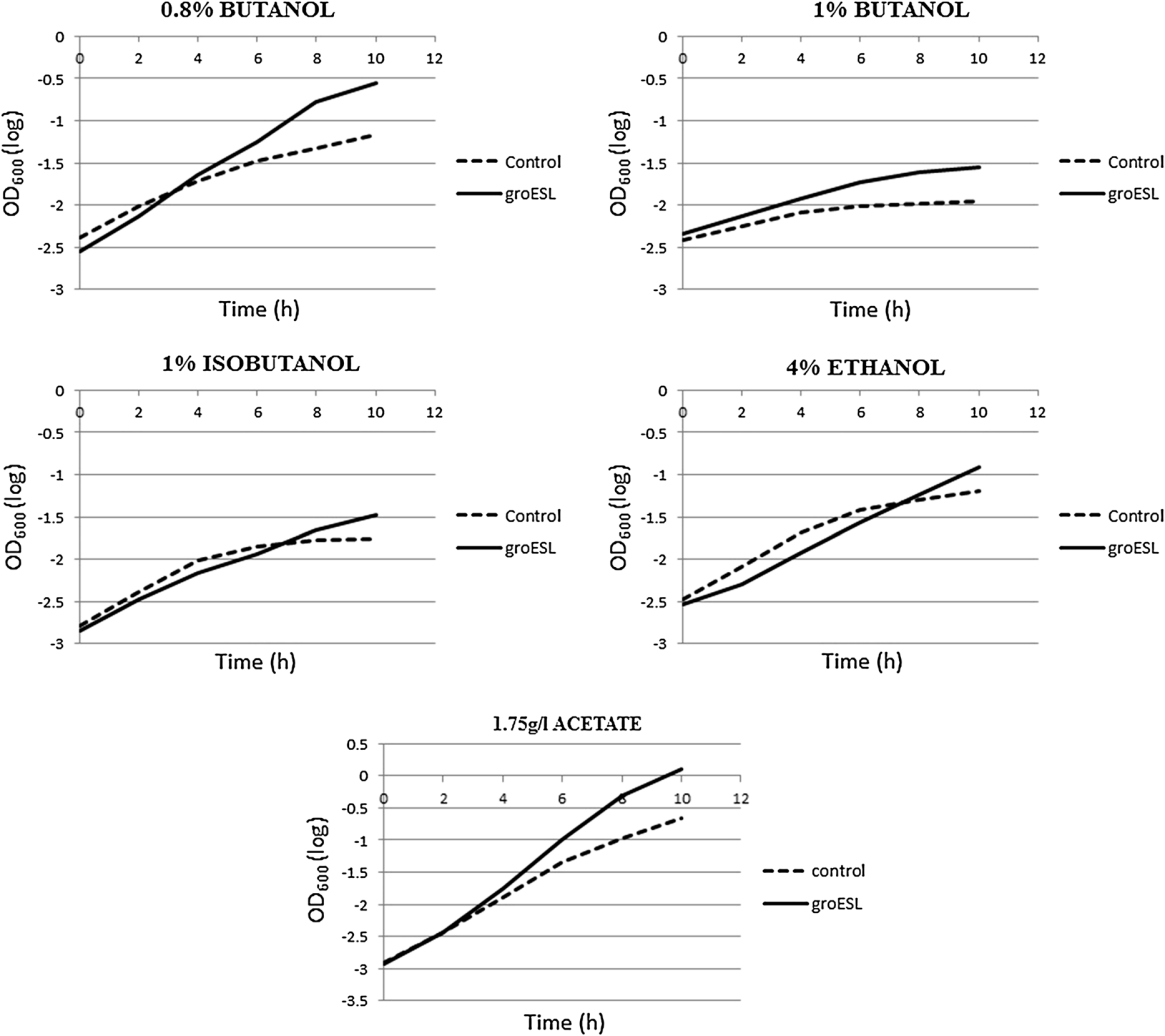
Growth curve of control and *groESL* with the challenge of different stressors

*E. coli* strain BL21 transformed by *groESL* was tested to increase the tolerance of *E. coli* to various stressors. *E. coli* strain BL21 transformed by the pF1A T7 Flexi harboring the barnase free vector was used as control, throughout these experiments. Both strains were challenged in the presence of 0.8 % butanol, 1 % butanol, 1 % isobutanol, 4 % ethanol, and 1.75 g/l acetate, separately. The impact of different stressors on cell growth was examined after 10 h of treatment. The optical density (OD) revealed that the overexpression of *groESL* enabled a significant increase in growth after 10 h for all stressors compared to the control (Fig. 4). Under solvent stress, the control strain demonstrated an exponential growth phase much shorter than the transformed strain. Transformed strain reached consistently higher optical densities and maintained higher cell concentrations over the control strain for the period-examined (Fig. 4). This confirms the ability of *groESL* to induce tolerance in the transformed strain, which results in prolonged exponential phase. On the other hand, the control strain showed less tolerance to stressors by reaching the stationary phase earlier.

### Effect of *groESL* overexpression on tolerance to butanol

**Fig. 5:**
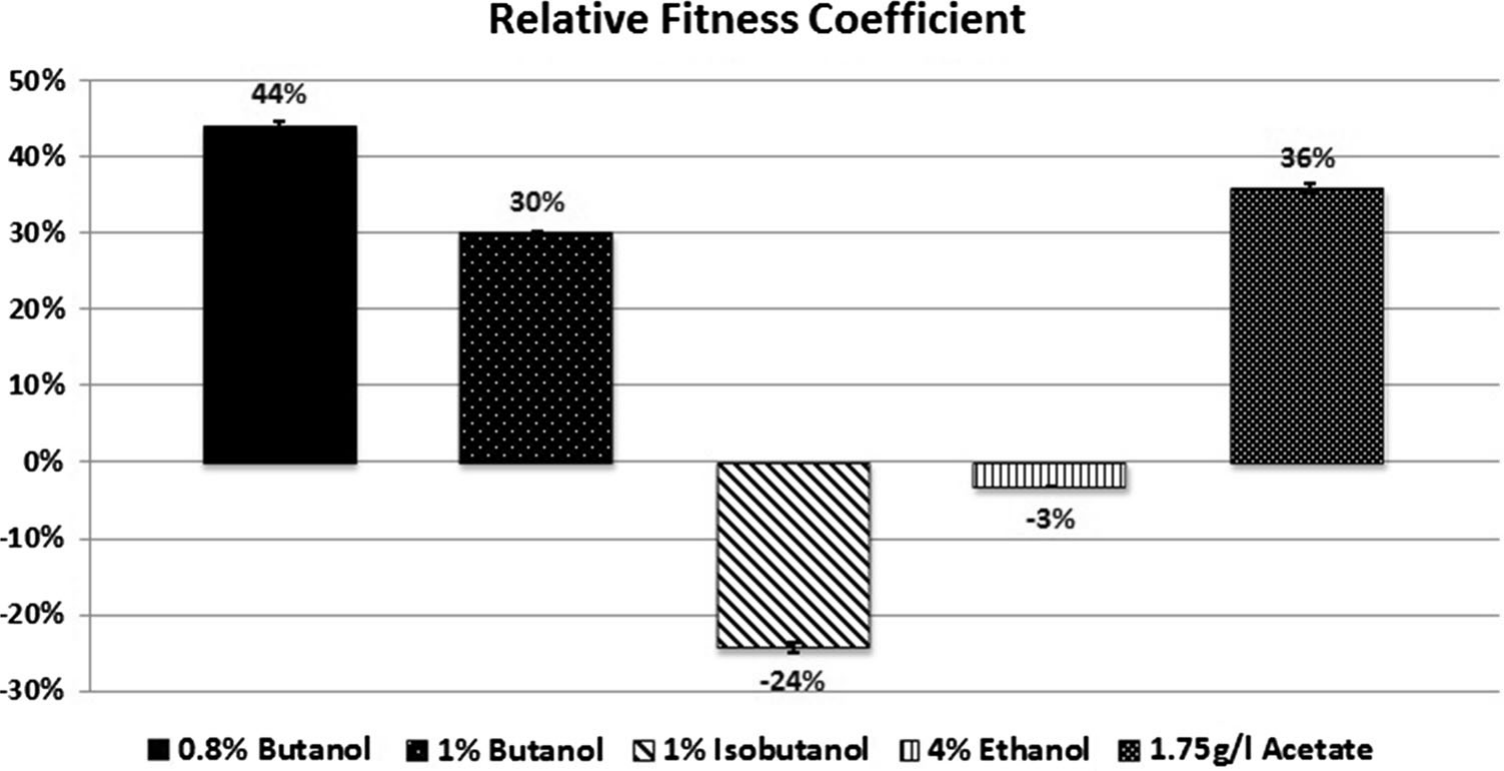
Relative fitness coefficient of *groESL* with the challenge of butanol, isobutanol, ethanol, and acetate stressors. *Error bars* indicate standard deviation between replicate data

**Fig. 6:**
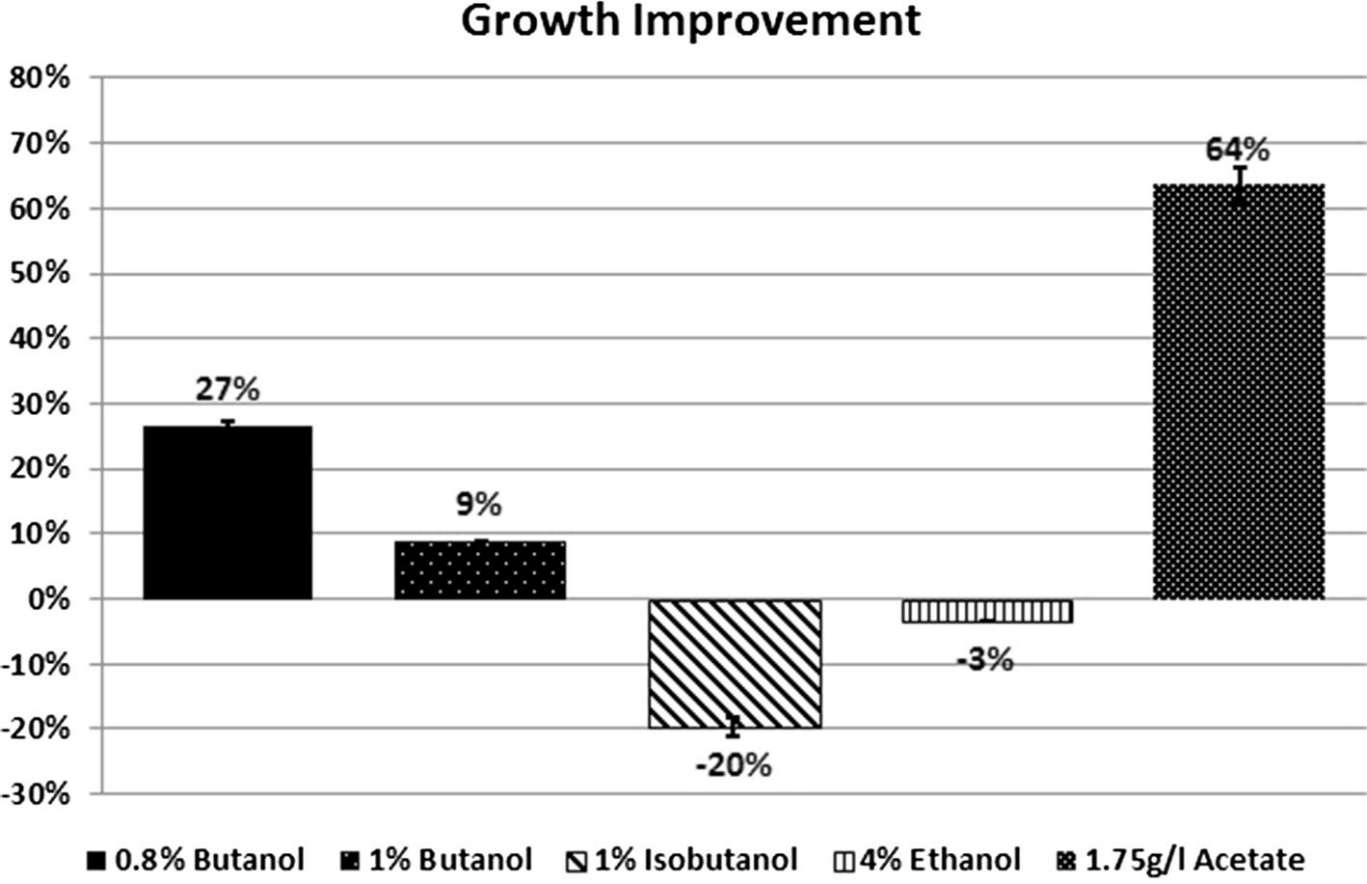
Growth improvement of the transformed strain with the challenge of butanol, isobutanol, ethanol, and acetate Stressors. *Error bars* indicate standard deviation between replicate data

**Fig. 7:**
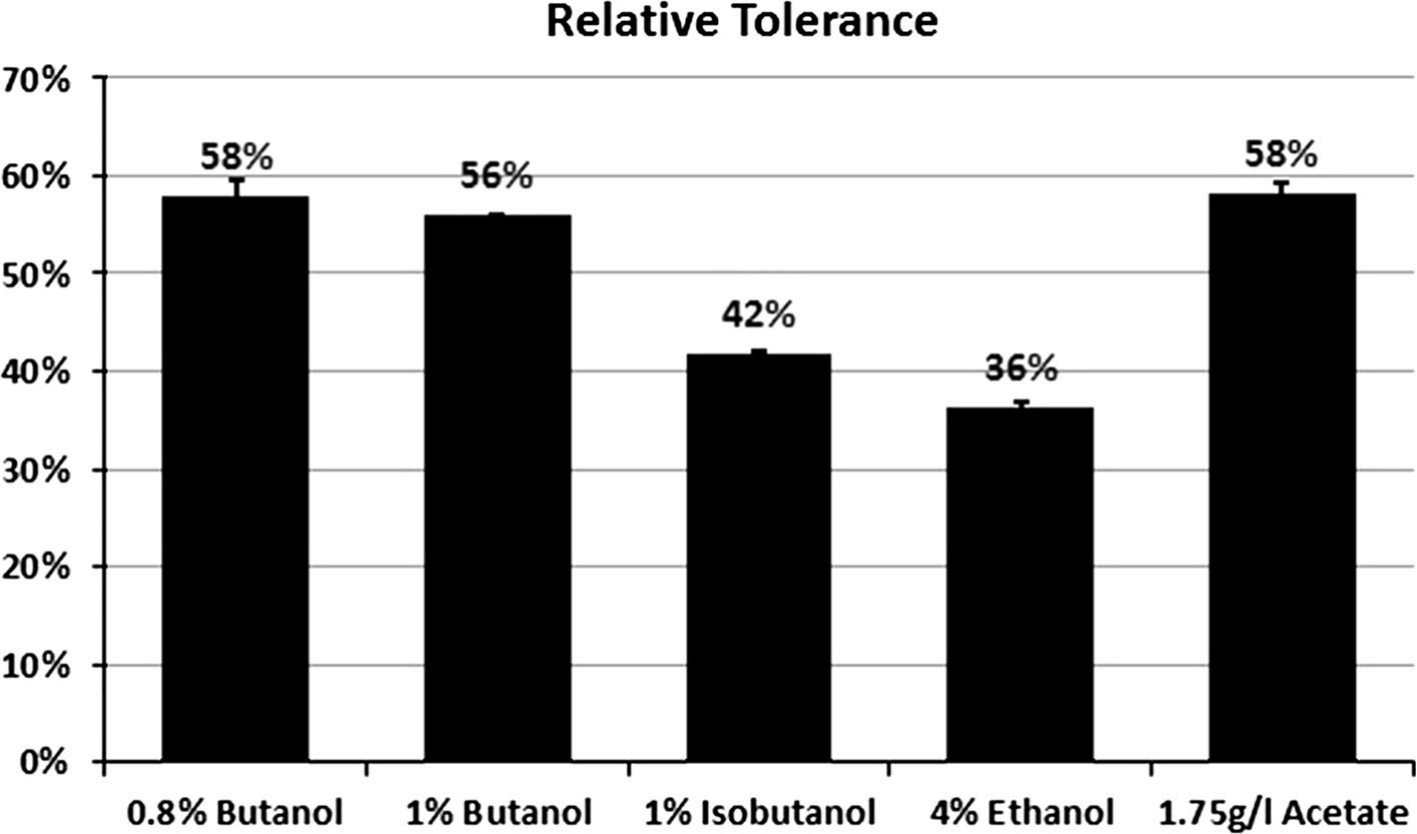
Relative tolerance of *groESL* with the challenge of butanol, isobutanol, ethanol, and acetate stressors. *Error bars* indicate standard deviation between replicate data

The kinetic parameters were calculated to determine the increase in stress tolerance. The ratio between the specific growth rates of the strain of interest relative to the control strain under each stress condition was determined using the relative fitness coefficient “s” (Eq. 2, in “Materials and methods”). The Relative Increase in Fitness, “RIF”, is a parameter calculated to normalize the relative fitness of the overexpression strain in the presence of the stressor against any fitness defects/advantage exhibited by the strain in the absence of the stressor. Positive values of RIF represent a net increase in growth rates in the presence of the stressor. A Student’s *t* test analysis (*p* value <0.05) was used to assess significance of the aforementioned calculated kinetic parameters. At 0.8 % butanol, a significant increase in growth was found in the transformed strain compared to the control (Fig. 4). When grown without solvent stress, the transformed strain and the control strain performed comparably in terms of growth. The fitness of the transformed strain was significantly increased with relative fitness coefficient of 44 % compared to control strain (Fig. 5) and the inhibition of this strain reduced to 47 % compared to 64 % in control strain, i.e. the transformed strain yielded 27 % growth improvement (Fig. 6). The percent tolerance relative to unchallenged culture was estimated at the challenge level and sample time (Eq. 4). The relative tolerance (% RT) of strain compared to the control strain was estimated (Eq. 5). The percent of butanol tolerance in the transformed strain was significantly increased to 33 % compared to 14 % in control after 10 h of exposure to 0.8 % butanol with relative tolerance of 58 % (Fig. 7). At 1 % butanol, a significant increase in growth was found in the transformed strain compared to the control (Fig. 4). The fitness of the transformed strain was significantly increased with relative fitness coefficient of 30 % compared to control strain (Fig. 5) and the inhibition of this strain reduced to 74 % compared to 81 % in control strain, i.e. the transformed strain yielded 9 % growth improvement (Fig. 6). The percent of butanol tolerance in the transformed strain was significantly increased to 8 % compared to 3.5 % in control after 10 h of exposure to 1 % butanol with relative tolerance of 56 % (Fig. 7). In agreement of these results, Tomas et al. (2003) showed that synthetic overexpression of *groESL* in *C. acetobutylicum* imparts solvent tolerance with 85 % reduction in growth inhibition and leads to prolonged and enhanced growth, metabolism, and solvent production by up to 40 %. In addition, *groESL* overexpression was shown to increase tolerance to butanol in *L. paracasei* and *L. lactis* (Desmond et al. 2004). Moreover, overexpression of the *E. coli* *groESL* proteins improved tolerance to a variety of toxic solvents, apparently in a solvent-agnostic manner (Zingaro and Papoutsakis 2012a). Heterologous HSPs have also been used to improve organic solvent tolerance in *E. coli* (Okochi et al. 2008).

### Effect of *groESL* overexpression on tolerance to other solvents

Cell cultures were challenged using isobutanol and ethanol, separately in the nutrient media to evaluate the effect of *groESL* overexpression on tolerance to those stressors, and all parameters were calculated. In the presence of 1 % isobutanol, using the kinetic parameters depend on the measured maximum specific growth rate (*µ*_*i*_) of each strain, the fitness of the transformed strain was reduced with relative fitness coefficient of 24 % compared to control strain (Fig. 5) and the inhibition of this strain increased to 65 % compared to 54 % in control strain, i.e. the control strain yielded 20 % growth improvement (Fig. 6). However, using the kinetic parameters depend on the measured growth after a period of time, the percent of isobutanol tolerance in the transformed strain was significantly increased to 12 % compared to 7 % in control after 10 h of exposure to isobutanol with relative tolerance of 42 % (Fig. 7). Similar results were shown with ethanol; in the presence of 4 % ethanol, using the kinetic parameters depend on the measured maximum specific growth rate (*µ*_*i*_) of each strain, the fitness of the transformed strain was decreased but not significant with relative fitness coefficient of 3 % compared to control strain (Fig. 5) and inhibition of this strain reduced to 60 % compared to 58 % in control strain, i.e. the control strain yielded 3 % growth improvement (Fig. 6), while using the kinetic parameters depend on the measured growth after a period of time, the percent of ethanol tolerance in the transformed strain was significantly increased to 22 % compared with 14 % in control after 10 h of exposure to ethanol with relative tolerance of 36 % (Fig. 7). While the trend is an increase in toxicity with an increase in solvent hydrophobicity, the mechanism of toxicity varies with the length of the carbon backbone (Aono and Nakajima 1997; Rutherford et al. 2010). Most toxicity studies have proposed the cell membrane as the most affected target of organic solvents and a significant factor in adapting to the stress. Both long- and short-chain alcohols are known to cause stress by either desiccation (short) or by intercalating in the hydrophobic cell wall fatty acids (long) (Ingram 1986; Ingram and Buttke, 1984; Kabelitz et al. 2003; Rutherford et al. 2010) and may be critical factors in the robustness of a host microbe during fuel production. It was demonstrated that Gram-negative bacteria are generally much more resistant to increasingly polar solvents than Gram-positive prokaryotes (Inoue and Horikoshi 1991; Vermue et al. 1993). The abilities of the different alcohols to induce the heat shock response are proportional to their lipophilicities: the lipophilic alcohol isobutanol is maximally inductive at about 0.6 M, whereas the least lipophilic alcohol, methanol, causes maximal induction at 5.7 M (Meyer et al. 1995).

### Effect of *groESL* overexpression on tolerance to acetate

Cross-tolerance between acetate and n-butanol stress have been identified previously in *C. acetobutylicum* (Nielsen et al. 2009; Alsaker et al. 2010), and thus was included as a test condition here. The effect of acetate stressor on the cells was studied using 1.75 g/L prior to growth assay. A significant increase in growth rate was found in the transformed strain compared to the control strain as shown in Fig. 4. Using the kinetic parameters depend on the measured maximum specific growth rate (*µ*_*i*_) of each strain, The fitness of the transformed strain was significantly increased with relative fitness coefficient of 36 % compared to control strain (Fig. 5) and inhibition of this strain reduced to 14 % compared to 38 % in control strain, i.e. the transformed strain yielded 64 % growth improvement (Fig. 6), while using the kinetic parameters depend on the measured growth after a period of time, the percent of acetate tolerance in the transformed strain was significantly increased to 72 % compared to 30 % in control after 10 h of exposure to acetate with relative tolerance of 58 % (Fig. 7). In agreement of this result, HSP genes were shown to be up-regulated upon carboxylic acid (butyric and acetic) stress and *groESL* appears to be commonly up-regulated upon butanol and acetate stresses (Alsaker et al. 2010).

## Conclusion

Heterologous overexpression of *groESL* chaperone system from *C. acetobutylicum* was successfully employed on *E. coli* in order to increase its tolerance to several toxic stressors. Our results show that heterologous overexpression of *groESL* chaperone is a useful and efficient approach for developing butanol tolerant strain of *E. coli* to be a basic strain for butanol production.
